# Genomic Variation and Diversification in Begomovirus Genome in Implication to Host and Vector Adaptation

**DOI:** 10.3390/plants10081706

**Published:** 2021-08-19

**Authors:** Deepti Nigam

**Affiliations:** Plant Pathology and Plant-Microbe Biology Section, School of Integrative Plant Science, Cornell University, Ithaca, NY 14853, USA; dsinghbioinfo@gmail.com

**Keywords:** begomovirus, satellite, SNP, diversity, adaption, host range

## Abstract

Begomoviruses (family *Geminiviridae*, genus *Begomovirus*) are DNA viruses transmitted in a circulative, persistent manner by the whitefly *Bemisia tabaci* (Gennadius). As revealed by their wide host range (more than 420 plant species), worldwide distribution, and effective vector transmission, begomoviruses are highly adaptive. Still, the genetic factors that facilitate their adaptation to a diverse array of hosts and vectors remain poorly understood. Mutations in the virus genome may confer a selective advantage for essential functions, such as transmission, replication, evading host responses, and movement within the host. Therefore, genetic variation is vital to virus evolution and, in response to selection pressure, is demonstrated as the emergence of new strains and species adapted to diverse hosts or with unique pathogenicity. The combination of variation and selection forms a genetic imprint on the genome. This review focuses on factors that contribute to the evolution of Begomovirus and their global spread, for which an unforeseen diversity and dispersal has been recognized and continues to expand.

## 1. Introduction

Circular, Rep-encoding single-stranded (CRESS) DNA viruses (phylum *Cressdnaviricota*) is the group of single-stranded DNA (ssDNA) viruses encoding a replication-associated protein (Rep) that appears to have originated from a common ancestor [1]. Plant infecting CRESS DNA viruses are categorized into the families *Geminiviridae* and *Nanoviridae* [2]. Begomoviruses (BGVs) belong to the largest known genus of ssDNA viruses (the genus *Begomovirus* represents 88% of the family *Geminiviridae*), and they are responsible for a substantial amount of crop loss worldwide [3,4]. They are efficiently spread by a polyphagous whitefly vector, i.e., *Bemisia tabaci* (a collection of biotypes), to a broad host range, which encompasses both wild and cultivated plant species [5]. However, other species can also transmit BGVs, such as *Trialeurodes ricini* [6] and *Trialeurodes vaporariorum* [7]. Key symptoms of BGV infections include yellowing, inward curling of the leaves, and stunting of the plants, resulting in significant yield loss [8]. The genome can either be a single component (monopartite) between 2.5–3.1 kb or, in the case of some BGVs, two similar-sized components (bipartite), each between 2.6 and 2.8 kb [3]. Monopartite BGVs encode six proteins, viz. C1/Rep, C2/TrAP, C3/REn, C4, V2, and V1/CP [9]. Homologs are encoded in one of the genomic components of bipartite BGVs, firstly DNA A (in this case, termed AC1/Rep, AC2/TrAP, AC3/REn, AC4, AV2, and AV1/CP); secondly, DNA B (the additional component in bipartite species) encodes two additional proteins: the nuclear shuttle protein (NSP) and the movement protein (MP).

The two genomic components of bipartite BGVs are denoted as DNA-A and DNA-B. These components share no significant sequence identity, excluding an intergenic region (IR) (size of ~200 nucleotides). The IR comprises the replication origin (ori), a conserved stem-loop structure with the conserved nonanucleotide TAATATT//AC and repeat sequences (iterons) that are explicitly recognized by the viral replication-associated protein, Rep [10]. The IR is vital for preserving the integrity of the bipartite genome, allowing both components to be replicated by Rep, which is known to confer a high specificity for their cognate ori [11].

Although, the mechanism by which segmented and multipartite genomes have emerged is uncertain, evidence suggests they may have derived by fragmentation of the genome of their non-segmented ancestors, with defective segments becoming virulent by complementation [12]. Specifically for BGVs, Briddon et al. [13] proposed that the DNA-B could have emerged from a satellite molecule captured by the monopartite progenitor of all BGVs. Perhaps, this combination offered greater flexibility to the monopartite ancestors, and accordingly, it was sustained over the evolutionary process.

The two main phylogenetic clades of BGVs are predominantly revealed based on their geographical distribution and genome organization: The Old World BGVs can be divided into African, Indian, Japanese, and Oceania, with a small number of strains falling outside these [13]. The New World BGVs are distributed into Central and Southern America [13]. The NW BGVs (~140 species) have a bipartite genome (DNA-A and DNA-B) with a few reported exceptions, while the OW BGVs include both bipartite and monopartite species, with a predominance of the latter (ca. 85%) [9]. A key difference between the New World (NW) and Old World (OW) BGVs is that the latter has an extra small open reading frame (ORF) that leads and moderately overlaps the CP gene, termed AV2/V2 or “precoat” gene [14]. The OW BGVs are recognized as more ancient and diverse than the NW BGVs.

Vectors are imperative in spreading viruses from infected plants to healthy plants through various transmission strategies, including transovarial mode [15]. More than 320 species of BGVs are known to be transmitted by *B. tabaci* (Hemiptera: Aleyrodidae), a cryptic species complex that includes more than 44 morphologically indistinguishable species [16,17,18]. Accordingly, the potential permutations of interactions in nature could be over 320 species of BGVs × more than 44 putative cryptic *B. tabaci* species × 1000 s of crop species and varieties [17,19,20]. Consequently, BGVs are considered fast-evolving DNA viruses due to the global expansion in the population, dispersal of their whitefly vector, and the worldwide movement of plant materials, usually driven by human movements [19]. Previous studies have shown the differences in virus transmission efficiency in the races of *B. tabaci* isolated from OW and NW geographical regions [20]. *B. tabaci* is a complex of at least 39 morphologically indistinguishable biotypes that are challenging or unmanageable to discriminate based on their morphology. For example, *B. tabaci* B or Middle East-Asia Minor 1 (MEAM1), which originated in Middle East-Minor Asia, and *B. tabaci* Q or MED, which originated in the Mediterranean region, are the two utmost invasive and destructive whiteflies [16]. *B. tabaci* B and Q (basically B and Q) vary in feeding behavior, virus transmission efficiency, host range, endosymbionts, and insecticide resistance. However, both B and Q enormously damage plants by feeding on phloem tissue and transmitting BGVs [21]. In the past 30 years, both biotypes have dominated many countries worldwide and banished some native cryptic biotypes. A recent study revealed that MED populations had a higher level of genetic variation with multiple invasions than MEAM1. Molecular genetic methods, such as mitochondrial cytochrome oxidase I (mtCOI) [22] and nuclear (microsatellite) DNA [23], have been used to investigate the ecological and evolutionary aspects of biological invasions and their concurrent impacts on the genetic structure and variation of an invasive species [24].

BGVs are often associated with DNA satellites, designated beta- and alphasatellites, promote vector–host interaction, suppress host defense, and support symptom development [25]. Rolling circle amplification (RCA) has revolutionized the diagnosis and genomics of BGVs and their associated satellites. This success is mainly due to the accessibility of RCA using φ29 DNA polymerase, a technique that allows the amplification of ssDNA viral genomes without any prior knowledge of nucleotide sequences [21,22]. RCA has also enhanced the detection of many small noncoding DNA satellites that are a quarter of the size of their cognate helper BGV genomic components [23,24]. Deltasatellites have recently been proposed for these satellites [26]. The association of betasatellites (also called symptom-modulating satellites) with the majority of the Old World monopartite BGVs and their unrestrained trans-replication by diverse helper BGVs have made them a severe threat to the agro-economy [27,28,29,30]. Alphasatellites are self-replicating circular single-stranded DNA molecules (size 1.3 kb to 1.4 kb) and requisite helper viruses for their movement inside the host plant and vector transmission. Their exact function is not well-known [27,28,29]. However, in another study, the function of alphasatellites in disease severity via affecting the virulence of the helper virus has been demonstrated [30,31,32]. Betasatellites are circular single-stranded DNA molecules (size 1.3 kb) and are entirely reliant on their helper viruses for replication, encapsidation, movement, and vector transmission [33,34]. Betasatellites are often associated with symptom development, disease diversification, and increased accumulation of viral nucleic acids in the host [35,36].

BGVs from the NW and OW geographical regions are known to be genetically divergent. Phylogenetic analyses suggested independent segregation, where the OW BGVs clade displayed a greater genetic diversity [37]. Diverse environmental factors frequently influence virus transmission and tritrophic interaction between plant–vector–virus, where the vectors are vital mediators. Virus replication in both vectors and plants enforces an evolutionary pressure over the virus genome. For example, viruses jump in different hosts and experience robust and strict adaptive selection as they intensify their fitness for the new niche. Therefore, the host might act as a primary driver of the longer-term evolution of viruses. Based on the same hypothesis, Simmonds et al. proposed a “niche-filling model” and highlighted the role of host interactions in shaping virus evolution [38]. Some non-cultivated plant species, especially of the families *Malvaceae*, *Euphorbiaceae*, *Fabaceae,* and *Solanaceae*, are identified hosts of BGVs [39].

Genomic variation, evolution, and adaptation of the viruses to distinct hosts are reconciled by the combinative effect of genetic factors in their genome and selection pressure imposed by the host [40,41,42]. Besides, different host species may play an essential role in the standing genetic variability of BGV populations [38]. Mutations are the leading source of variation for most BGV populations [43]. Selective pressures applied by the host play a critical role in shaping virus populations, and these populations are likely being selected for at both the protein and DNA or RNA levels [44]. Accordingly, the chronological mechanism of emergence for some mutational patterns (nucleotide and amino acid substitutions) over the virus genome is key for anti-viral defense. Recent studies based on computational analyses have allowed the identification of fractions of non-synonymous to synonymous substitutions to determine virus evolution (as diverging clades) [41,45]. Purifying selection diminishes the volume of non-synonymous substitutions before they arise or are fixed in the genome and favors the fixation of those involving adaptive benefits. In contrast, synonymous substitutions are more likely to be maintained [46]. Attributing them to the small genome size with a high potential for genomic variation (due to mutation and recombination), BGVs are attractive models for studying the evolutionary and ecological factors driving their emergence [47]. Substitution rates (or μ) of whitefly vectored BGVs have been described to be equally high as those of ssRNA viruses [48,49], and positive selection pressure on mutations or the products of recombination events plays a crucial role in BGV evolutionary dynamics [50,51]. Regulatory mechanisms of BGVs and RNA viruses promote host adaptation; the betasatellite silencing suppressor βC1 avoids excessive inhibition of antiviral pathways and cell toxicity through autophagy activation [52]; *Cucumber mosaic virus* (CMV) silencing suppressor 2b and its interacting partner ARGONAUTE 1 (AGO1) is antagonized by the viral CP and 1a [53,54]; and the regulated proteolysis of the Plum pox virus (PPV) P1 modulates the HCPro silencing suppressor activity to promote the long-term virus fitness [55].

DNA/RNA methylation is also an essential epigenetic modification that could affect plant immunity, virus adaptation, and evolution [56,57]. Independent studies have disclosed that geminivirus–betasatellite complexes are both robust inducers as well as targets for both post-transcriptional gene silencing (PTGS) and transcriptional gene silencing (TGS) and thus play a fundamental role in virus–host interaction [58,59]. To lower the host antiviral RNA silencing defense, the *βC1* protein encoded by several betasatellites can suppress PTGS. Moreover, epigenetic modifications of histones (ubiquitination, methylation) associated with the minichromosomal structure of monopartite capsicum-infecting BGVs have been shown to play a crucial role in virus–host interaction [58]. An excellent recent report investigated the incidence of BGVs adept at modulating plant immunity to enhance the fitness of their whitefly vector and diminish the performance of two non-vector herbivores [60]. They indicated that the βC1 proteins encoded by the satellites associated with *Cotton leaf curl Multan virus* (CLCuMuV) and *Tomato yellow leaf curl China Virus* (TYLCCNV) could interact with the transcription factor WRKY20 and thus stimulate a plant tissue-specific response against different herbivores. Consequently, satellite DNAs need further investigation, as they may be a key factor driving the diversification of begomovirus–satellite disease complexes.

Begomoviral proteins have been characterized for understanding the mechanism of symptom recovery [61,62], virulence, and host resistance [63,64]. Coat protein (CP) is a multifunctional protein due to its interaction with plants and vectors [65]. The CPs of all the whitefly-transmitted geminiviruses have one or more antigenic epitopes in common, suggesting that these could be determinants of vector specificity and that they play a leading role in virus transmission [66,67]. Recently, an *in silico* study showed a higher mean diversity in the *cp* gene of OW BGVs compared to the NW [68]. However, highly mutable amino acids have been identified in the CP of *Squash leaf curl China virus* (SLCCNV) [69], which did not alter their fitness in the host plant but rendered the virus more competitive for certain species of whiteflies.

Although several techniques ranging from conventional methods to molecular advances have been implemented to control geminiviral infections, the success has been limited due to synergistic virus infections. CRISPR-Cas (Clustered, regularly interspaced short palindromic repeats, CRISPR, associated protein), a bacterial adaptive immune strategy against interfering foreign nucleic acids, has emerged as effective genome editing technology that has been successfully applied in many organisms, including several plant species [70,71,72]. Nevertheless, rapid genetic variation and virus evolution evidence include the escapee characterization from CRISPR-Cas9 plants engineered to target BGV genomes. Ali et al. underlined a potential problem with the technique by determining the probability of virus escape from the CRISPR-edited plants [73,74,75]. However, virus escape from editing was also proven by Mehta et al., whose efforts to persuade resistance against *African cassava mosaic virus* (ACMV) in permanent transgenic cassava (*Manihot esculenta*) lines showed limited success [76]. Therefore, selecting targets within the viral genome is a crucial factor in conquering durable resistance. In this perspective, non-coding targets are more efficient over coding regions as they embed the key elements for virus replication and pathogenicity maintenance [77]. Furthermore, detecting the potential host factors involved in the resistance during plant-geminivirus interaction, multiplexed genetic engineering tools directing multiple targets, and targeted deletion in viral genomes can assist in developing disease-free plants and counteracting the emergence of CRISPR-resistant BGVs.

*Tomato yellow leaf curl virus* (TYLCV) has the highest host range across BGVs and has been discovered in 49 species belonging to 16 different plant families [78]. In the tomato plant, various sources of resistance to TYLCV have been recognized and employed to produce resistant cultivars. Despite broad efforts to control TYLCV by deploying resistance in the field, new variants efficient in overcoming resistance have continuously emerged, and TYLCV remains the most widespread and damaging virus both in tomato and pepper crops. Apparently, wild tomato species, like *Solanum pimpinellifolium*, *S. peruvianum*, *S. chilense*, *S. habrochaites,* and *S. cheesmaniae,* are resistant to TYCLV and other BGVs. Resistance genes, such as *Ty-1* to *Ty-6* [79,80], from these wild relatives have been repeatedly backcrossed into cultivated tomato varieties, leading to the improved resistance to the virus, but they were never 100% resistant [81]. Resistance-driven selective pressure combined with the high evolutionary capacity of TYLCV might have contributed to the unique evolution of TYLCV. Therefore, more cohesive advancements that complement host resistance are essential for the successful control of TYLCV.

## 2. Discussion

BGVs have become the most devastating group of plant viruses in tropical and subtropical regions of the world. The current emergence of BGVs is noteworthy, as these viruses have been co-evolving with their dicotyledonous plant hosts for ages. The plant hosts and varieties grown will influence virus diversity through selecting for viruses and vector populations. Agricultural growth has been suggested as one of the leading causes, together with expansions in populations of their vector *Bemisia tabaci*, moderately due to the worldwide spread of the more prolific B-biotype with new diseases and associated epidemics. 

The fecundity of different *B. tabaci* populations vary significantly on diverse hosts [82], and cultivated crop fluctuations might result in discrete changes in vector abundance. For instance, the increased cultivation of cotton, soybean, and other horticultural crops in Latin America in the 1970s led to greater *B. tabaci* populations and subsequent BGV disease [39]. The main driving force for the destructive cassava mosaic pandemic that has spread quickly in East Africa since the late 1980s [83] shows to be an interaction between virus strains, vector populations, and host genotypes rather than a single factor [84]. The fecundity of *B. tabaci* spreads drastically on cassava plants infected with the recombinant EACMV-[UG], an *East African cassava mosaic virus* Uganda (Uganda variant) to a much higher-population density on the restricted green areas of severely affected leaves and an increased migration rate of infective adults.

One other fundamental area that needs clarification is the role and mode of interaction of the newly discovered circular ssDNA satellites with each other, their helper viruses, and their role in BGV epidemiology. These DNA satellites share no significant sequence homology with their helper BGV sequences and are of various types. The epidemiological role of DNA-β satellite molecules seems to be in extending the host range of BGVs. For example, at least five diverse BGV species, including *Papaya leaf curl virus*, can cause cotton leaf curl disease in Pakistan but only when associated with a particular DNA-β molecule [85].

Little is known about the selection pressures that seem to operate and drive BGV evolution towards increased virulence and an extended host range. However, the genomes of BGVs show extreme plasticity, leading to rapid evolution in response to changing cropping systems. Genetic factors determining virulence, host adaptation, and suppression of defense responses are under positive selection [86,87]. In the perspective of naturally distinct hosts and vectors, BGVs may face differential selection pressure to maintain functionality [46]. Furthermore, geographically distinct host and vector genetic diversity enforce various selective constraints [58,86]. Every combination of a host and virus is unique, and assisting different variant selection provides new host adaptation, a new strain, species emergence, and ultimately host range extension. Irrespective of human or plant viruses, experimentally validated co-evolving amino acids are associated with a host shift [88,89]. It is known that a change in a set of a few amino acids of viral proteins (co-evolutionary) can lead to a change in the host infectivity range. However, recent progress in analyzing mutation libraries and the interaction between viral three-dimensional protein structures and host factors can enhance co-evolutionary amino acid discovery and our understanding of the viral evasion landscape [90,91]. Some studies have proven the relation between sequence amino acid co-variation in viral determinants and host adaptation [92,93,94]. Such modification occurs over a genetic adaptation process that overcomes viral entry and replication barriers in a new cellular environment.

Previous findings indicate that additional virus-induced driving forces for BGV epidemics might be the alteration of plant biochemistry so that infected plants emit volatiles as vector attractants, alter feeding behavior, enhance vector fertility [95], and allow increased virus acquisition [96]. These interactions could have the consequence of increasing the robustness of the virus population. Biological and genetic studies to elucidate such interactions are a high priority for future research. Additionally, experimental study should be combined with mathematical modelling studies [97], which offer prospects for dissecting and incorporating the various layers of interaction and exploring the consequences for virus epidemiology [98,99,100].

**Figure 1:**
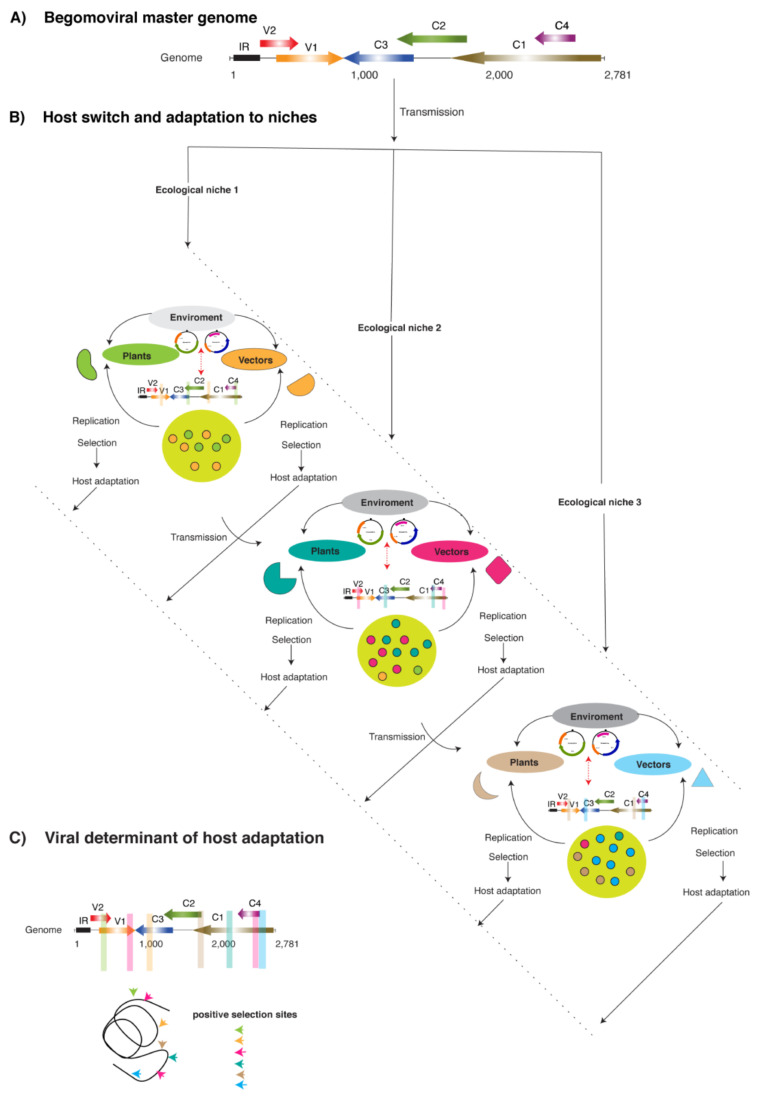
Model for Begomoviral genomic diversity and adaptation. (**A**) A Begomoviral master genome. Begomovirus (BGVs) encodes 6–7 proteins (V2 and V1) from the sense strand and C3, C2, C1, and C4 in the antisense strand. (**B**) BGVs may be transmitted by their vector (*B. tabaci*) in genetically diverse hosts (species, cultivars, or landraces). Due to various environmental climates and geographical niches, the genotype of the plant and vector may differ. Virus replication and selection within-host is a continuous process. During this process, the interaction of begomoviral proteins with pro-viral and antiviral proteins (host and vector) regulates the balance between variation and selection, leading to the selection of the fittest, most adapted strains. Vectors contribute to selection by transmitting the virus in new plant species or different genotypes/cultivars of the same species. Some BGVs retain a satellite called DNA β (betasatellite), and this interaction is called begomovirus–betasatellite complexes (red dotted arrows). Betastaellites depend on the helper virus for their replication and spread within and between hosts. Selection pressure enforced on a virus genome by a given environment will alter the virus population, excluding less fit entities. Mutations that offer a beneficial advantage are probable to be fixed in the genome. Some mutations generated in alternates hosts (from different niches) might break resistance and expand the host range. (**C**) During the evolutionary process, beneficial mutations (non-synonymous), including sites under positive selection, differentially accumulate in different viral proteins. They might contribute to fitness by enhancing stability, transmission, replication competence, escape from immunity, suppression of immune responses, or a combination.

BGVs co-evolve with their hosts and vectors in diverse environments and face selection pressure for any host–vector combination to maintain genomic organization and protein functions that facilitate vector transmissibility, replication, and movement [69,101]. Based on the same hypothesis, we presented a model for BGVs evolution via an example of a typical BGV master/founder genome (Figure 1A). While hosts from different niches favor the better adapted variants to replication and movement, vectors select them based on transmission efficiency before or after adaptation to a particular host (Figure 1B). Additionally, the trans-replication of betasatellites by different BGVs may trigger diversity in the BGV genome by acquiring homologous iteron-like motifs [102] (Figure 1B). The previous study based on phylogenetic analyses has shown the segregation of betasatellites according to their host and geographic origin [103]. These results strongly encourage the concept of coadaptation of betasatellites with their corresponding helper BGVs. Accordingly, genetic plasticity in key segments of the BGV genome must sustain functionality in genetically diverse hosts, vectors, and environments. Tolerance for new mutations may provide the robustness required for generating selection diversity to identify variants with a competitive advantage. Thus, due to the repeated cycle of virus replication in a host plant, vector transmission and selection may lead to host adaptation. In the present model, mutations get fixed in begomoviral proteins that are determinants of host adaptation and vector transmission (Figure 1C).

Investigation on the molecular diversity of BGV populations prerequisites to focus the population rather than ‘molecular’ level [104], as simply determining the number of different molecular sequences present in a host plant, crop or region, is insufficient to track evolutionary change and determine the influence of factors such as the introduction of host-plant resistance, or changes in cropping system. Also, there is still a lack of evidence on the exact rate of virus variants and inevitably there will be biases in the current information of virus diversity. Diagnostic practices, such as polymerase chain reaction (PCR), are selective even when degenerate BGV PCR primers are used. Besides, many reports of gene or genome function have dealt only with the properties of infectious clones of one sequence. In the field, the biological function of the virus may depend on the interaction between a ‘swarm’ of variant sequences upon which selection acts [105]. Recent advances in DNA synthesis have allowed the establishment of a synthetic genomics framework that can significantly accelerate the biological characterization of BGVs and their satellites [106].

In summary, these few illustrations highlight the practicality and importance of beneficial mutations in the plant viral genome, providing acquaintance behind the resistant outbreak. Therefore, understanding the influence of the evolutionary direction of virus populations is vital for developing more durable strategies to control begomoviral diseases in crop fields. The abundance of these mutations is manifold, and it is secure to accept that many of the viral determinants in BGVs have not yet been identified. In conjunction with enhancements of practical genome engineering approaches, novel viral functions will be discovered. 

## 3. Conclusions

Genetic determinants facilitate virus variation, evolution, and adaptation to diverse hosts and environments. The potential of BGVs to evolve rapidly by acquiring genes from other BGVs or viruses of different genera enhances further complications. Consequently, a commencing point is mutations (nucleotide substitutions, insertions, deletions), recombination, and reassortment (in segmented viruses). While these mutations may occur randomly, selection separates beneficial from unfavorable and neutral mutations. Selection is enforced by the host, the environment, and their interaction. Mutations that provide a beneficial advantage are more likely to be fixed in the genome and accumulate to higher than random frequencies in areas of the genome that contribute to robustness by enhancing stability, transmission, replication competence, escape from immunity, suppression of immune responses, or a combination. Unquestionably, there will be no specific solution to monitoring these epidemics, and the human impact on BGV evolution can only be minimalized by constraining or altering some practices that have led to the advent of these viruses and their vector populations.

## 4. Prospects

Our review illustrates that we are only beginning to comprehend the tripartite interactions between BGVs, vectors, and host plants. The perpetual resurgence of new recombinant strains of TYLCV or any other BGV species might lead to resistance breaking, efficient vector transmission, and expansion of the host range, thus being a significant threat to crop production and disease management. In the future, it would be imperative to study a multidisciplinary approach, such as the combined study of host and geography, vector/human-mediated dispersal, and molecular interaction fundaments of begomovirus-satellite disease complexes, for the in-depth understanding of their expanding virosphere. It would help to determine the viral determinants of vital importance, broader infectivity, and a potential antibody target.
